# Knowledge and attitudes towards HIV vaccines among Soweto adolescents

**DOI:** 10.1186/1756-0500-1-76

**Published:** 2008-08-29

**Authors:** Guy de Bruyn, Nokuthula Skhosana, Gavin Robertson, James A McIntyre, Glenda E Gray

**Affiliations:** 1 Perinatal HIV Research Unit, University of the Witwatersrand, Johannesburg, South Africa

## Abstract

**Background:**

To explore adolescent HIV risk perception, HIV vaccine knowledge, willingness to participate in future HIV vaccine clinical trials, and the factors that influence willingness to participate among high school students in Soweto, South Africa, we recruited school-going youth through randomly selected local high schools. All pupils within the selected schools from whom parental consent and child assent could be obtained were eligible for participation. A self-administered, facilitated questionnaire was completed by all participants.

**Findings:**

Perception of adolescent HIV risk was high. Some misconceptions regarding vaccine research were common, particularly regarding placebo and potential eligibility criteria for prophylactic vaccine trials. Of 240 responses to the willingness item, 84 (35%) indicated they were "probably willing" and 126 (52.5%) that they were "definitely willing to participate". There were no significant differences in willingness by gender, age, school grade, or institution. Factors that were rated as "very important" in determining willingness included receiving current information about HIV research [n = 201 (88.9%)], doing something to honour people who have HIV or have died of AIDS [n = 168 (70.9%)], getting free counselling and testing [n = 167 (70.5)], that participants may receive some protection against HIV infection from the vaccine [n = 160 (70.2%)], and improving motivation to avoid risky behaviour [n = 134 (59%)].

**Conclusion:**

Soweto school-going youth report high degrees of willingness to participate in HIV vaccine trials. This may be related to the high levels of adolescent HIV risk perception. Whether hypothetical willingness translates to participation will await data from adolescent HIV vaccine trials.

## Background

In South Africa, adolescents are extremely vulnerable to HIV infection. Worldwide, about half of all new HIV infections among adults occur in people under the age of 25 [1]. Each day, 7000 people between the ages of 15 and 24 are infected with HIV worldwide, resulting in 2.6 million new infections per year, 1.7 million of which occur in Africa [2]. Similarly, the highest rates of HIV infection in South Africa occur among young people [3]. A national household survey conducted in 2002 estimated that about 10% of 15–24 year old South Africans are living with HIV [4]. The prevalence rate was 4.8% among boys and 15.5% among girls of the same age group in a second national survey conducted in 2004 [5]. While South African adolescents have widespread exposure to HIV prevention messages and knowledge of HIV aetiology [6], perception of vulnerability to HIV remains low [7,8]. High levels of risky sexual behaviour among teens are exemplified by, for instance, a high teenage pregnancy rate. The 2004 survey estimated a pregnancy rate of 15.5% among 15–19 year old South African girls [5]. In light of these behaviour challenges, an HIV vaccine will be an important tool for protecting adolescents and therefore testing promising candidate vaccines amongst adolescents is required.

Over the last two decades, candidate HIV vaccines have been studied in safety and efficacy trials involving healthy adult volunteers, and more recently infants and pregnant women have been included in early phase studies [2]. On the contrary, candidate vaccines have not been tested in adolescents. Currently, phase I/II vaccine trials are being carried out with the adult population in Soweto. The United States Food and Drug Administration (FDA), one of the regulatory bodies involved in oversight of clinical trials of HIV vaccines, stipulates that a drug cannot be licensed for use in a population in which it has not been tested. For this reason, lack of adolescent participation in HIV vaccine trials will only delay access of this age group to a vaccine once it is available [9]. Willingness of adolescents to enrol in HIV vaccine clinical trials is therefore an important factor in planning such initiatives.

Prior studies indicate that willingness to participate in future HIV vaccine trials is contingent on vaccine knowledge and to hypothetical trial attributes [10,11]. Men who have sex with men (MSM), whose baseline knowledge of HIV vaccines was very low, became unwilling to participate with increased knowledge. However, other findings have not borne out such a relationship, with one study suggesting that high levels of knowledge regarding HIV vaccines may lead to the increased likelihood of participation [12], while another did not find a relationship between knowledge and willingness to participate [13]. Willingness to participate in a hypothetical vaccine clinical trial may not reflect willingness to participate in an actual trial [10,14]. Some of the factors, identified among adults, that would discourage participation in HIV vaccine clinical trials include mistrust of medical research, social harms, and concerns regarding vaccine safety and vaccine-induced HIV seropositivity [10,11,13]. Some of the perceived benefits of trial participation include risk reduction counselling, altruism, and possible protection from the vaccine.

Other challenges inherent in adolescent clinical trial participation include many socio-legal and psychological considerations. In particular, adolescents may lack cognitive maturity to weigh the pros and cons of clinical trial participation [15]. This is further complicated by the importance of peer pressure on decision making. Adolescent participation in clinical trials requires parental consent, potentially compromising the privacy and confidentiality of the participant. Another important consideration when planning adolescent participation in clinical trials is to build in flexibility that will accommodate the needs of the adolescents.

To begin to understand adolescent attitudes to these complex issues, and inform our future work with adolescents in HIV vaccine trials, we undertook a formative study examining attitudes towards such trials, potential motivating factors and hypothetical willingness to participate, among Soweto youth. There are varied meanings of 'adolescent'; in this research we used the psychological sense of the intermediate time of development between childhood and maturity, rather than in a legal sense referring to a specific age, although examined responses in relation to the specific age, given age would be an eligibility criterion in trials.

## Methods

We undertook a study of attitudes to HIV vaccine trial participation among high school students in Soweto from August 2005 to February 2006. This study was approved by the University of the Witwatersrand Human Research Ethics Committee (protocol number MO50101). Approval was also provided by each school head teacher and the Gauteng Department of Education. Consultations were held with school governing bodies prior to initiation of the survey within each school.

### Participants

A two-stage sampling procedure was used. The first stage sampling units were all 72 public high schools in Soweto. Ten schools were randomly selected. Based on our estimates of class numbers and response rates, we planned to enrol students at four schools; we oversampled from the first stage pool to provide for potential non-agreement to conduct the research at a selected school. The first four randomly chosen schools were approached regarding participation and all agreed. All pupils within the selected schools from whom parental consent and child assent could be obtained were eligible for participation.

### Measures

A self-administered, facilitated questionnaire was administered to all participants [see Additional File 1]. The questionnaire explored general HIV knowledge, perception of adolescent risk, knowledge of vaccine concepts, willingness to participate in future vaccine trials, perceived personal and social harms and benefits associated with participation as well as barriers and facilitators to participating in future HIV vaccine trials.

The measures administered were based and adapted from a similar survey conducted among high-risk populations in the United States [10]. The items were adapted for comprehensibility and local idiomatic language use, and piloted prior to use in the school setting. The items had not been validated in prior work in South Africa, to our knowledge.

### Sample size

The sample size was estimated based on an initial assumption that 20% of adolescents would be willing to enrol in an HIV vaccine trial. To compare the hypothesized value against an alternative proportion of 50%, with type 1 error rate of 0.05 and 90% power, we estimated that 23 respondents would be required within each stratum. We will therefore recruit a minimum of 25 children of each gender within each of three age strata: < 13 years, 13 – 14 years, and 15 – 18 years. The minimum sample size for the questionnaire portion of this study will therefore be 150 participants.

## Results

277 school-going youth (mean age 16.2 years; range 10 – 25; 53.1% female) provided assent or consent to participate, and if under 18, we also obtained written consent from a parent. The response rate to the survey is not known. Not all data fields were adequately responded to, resulting in missing data for some items.

### Perceptions And Attitudes To Adolescent Sexuality And HIV Testing

Of the 241 participants who responded to the question on HIV testing, 10% indicated that they have tested for HIV (Table 1). Of those who responded that they had not previously tested, almost half (46%) of the participants stated that a reason for not testing was that they believed that they were not at risk of HIV infection, while 44% stated the reason was that they were afraid to test for HIV. More than half of the participants believed that sexual debut in Soweto is as early as 9 years of age and 43% believed that 9 year olds were vulnerable to HIV infection. However, less than half of the participants believed that adults talk to children that age about HIV. The majority (57%) of participants believed that parents should give permission for their child's HIV test while most of the participants (84%) believed that parents should know the HIV status of their child.

**Table 1:** Questions Exploring Perceptions And Attitudes To Adolescent Sexuality And HIV Testing

	n (%)
I have tested for HIV	24 (10)
I do not think I am risk of HIV infection	110 (46)
I am afraid to get tested for HIV	107 (44)
My parents should know my HIV test results	198 (84)
Children age 9 are beginning to be sexually active	133 (56)
Adults talk to children between 9–10 about HIV	111 (47)
HIV is a threat to children from age 9	102 (43)
My parents should give permission for my HIV test	135 (57)

*Values may not add up to total number of participants as some fields had missing data.

### Trial Participation And Stigma

HIV stigma did not appear to impact on willingness to participate in a future trial. Most respondents felt that potential discrimination, being avoided as a result of trial participation, being perceived as at high risk of HIV, or being thought to have AIDS would not influence their decision to participate in trials (Table 2).

**Table 2:** Questions Exploring Stigma Resulting From HIV Vaccine Trial Participation

How important is stigma in making decisions about trial participation?	Very n (%)	Somewhat n (%)	Slightly n (%)	Not at all n (%)
I may be discriminated against at school if I volunteer	41 (18)	30 (13)	32 (14)	130 (56)
People may avoid me if I volunteer in a vaccine trial	40 (17)	25 (11)	41 (17)	132 (55)
People may think I have HIV or AIDS if I volunteer in a vaccine trial	26 (11)	37 (17)	37 (17)	136 (58)
People may think I am at high risk of HIV if I am in a study	44 (19)	32 (14)	38 (16)	117 (51)
People may not want to have sex with me if I am in a study	56 (23)	17 (7)	35 (15)	132 (55)

### Willingness To Participate In Future HIV Vaccine Trials And Associated Factors

Of the 240 responses to the willingness item, 84 (35%) indicated they were "probably willing" and 126 (52.5%) that they were "definitely willing" to join a study of a vaccine to prevent HIV. Combined, willingness to participate ('definitely' and 'probably') was 88.2% (95% confidence interval 84.1% – 92.3%, p < 0.00001). There were no significant differences in willingness by gender, age, school grade, or institution. Factors that were rated as "very important" in determining willingness included receiving current information about HIV research [n = 201 (89%)], that they would be doing something to honour people who have HIV or AIDS or have died of AIDS [n = 168 (71%)], getting free counselling and testing every six months [n = 168 (71%)], that participants may receive some protection against HIV infection from the vaccine [n = 160 (71%)], and would feel more motivated to avoid risky behaviour [n = 134 (59%)] (Table 3).

**Table 3:** Factors Influencing Willingness To Participate In HIV Vaccine Trials

	n (%) indicating factor was "very important" in relation to willingness
I would get free counseling and HIV testing at least once every 6 months	167/237 (70.5)
I would get current information about HIV research	201/226 (88.9)
I may feel more motivated to avoid risky behavior	134/224 (59.8)
I would get a small amount of money each time I came for a visit	58/238 (24.4)
I may get some protection against HIV infection from the vaccine	160/228 (70.2)
I would be doing something to honor people who have HIV or have died of AIDS	168/237 (70.9)

## Discussion

Sexual debut in Soweto was perceived to be very early, exposing adolescents to HIV. Ten percent of the participants in this study reported having previously testing for HIV. This figure perhaps reflects the difficulties in accessing HIV testing for Soweto youth. Comparative figures for testing uptake in Soweto are not available; however, national surveys report rates of prior testing of 30.5% among people 15 years and older [16]. Similar to other reports emanating from South Africa (6), youth in this believed that they were at risk of HIV acquisition. However, the proportion of participants who thought that they were not at risk is still high in light of the prevalence of HIV in young adults and the belief expressed that sexual debut is early. The high proportion of participants who believed that parents should give permission for the HIV testing and know the HIV status of their child indicates interdependence between parents and their children although this is not without problems. Parental involvement in adolescent trial participation will have to be thought out very carefully to balance the need for privacy and autonomous decision-making and the need to have adults oversee adolescent participation in vaccine clinical trials.

Soweto school-going youth report high degrees of willingness to participate in future HIV vaccine trials. Participants indicated that access to regular counselling and testing, current information, and potential impact on motivation to reduce risk behaviour were very important for determining willingness to participate. Whether hypothetical willingness translates to participation will await data from adolescent HIV vaccine trials. In the same vein, consideration given to factors that may encourage or discourage participation in such trials may differ from a hypothetical to an actual trial. However, that being said, the enormousness of the HIV and AIDS epidemic in South Africa has fostered a culture of participation in HIV/AIDS-related initiatives. In this regard, HIV stigma may be a minor consideration in such decisions. Some recent data suggest that HIV stigma may no longer be a major influence in decisions relating to HIV and AIDS [16]. Further, several international studies point out that willingness to participate in a hypothetical HIV vaccine study is strongly associated with being at high risk for HIV infection [10,17,18].

## Competing interests

The authors declare that they have no competing interests.

## Authors' contributions

GdB designed the study, analyzed the data, and drafted the manuscript. NS designed the study, carried out the field work, and helped draft the manuscript. GR participated in the study design and helped oversee the fieldwork. GG conceived of the study, participated in its coordination and helped to draft the manuscript. JM helped arrange funding. All authors read and approved the final manuscript.

## Supplementary Material

Additional File 1 Adolescent questionnaire v1 29 Dec 2004.pdf. Self-administered questionnaire utilized in this research. Click here for file
