# Supplementary material for: Knowledge and attitudes towards HIV vaccines among Soweto adolescents
Source: BMC Res Notes. 2008 Aug 29;1:76. doi: 10.1186/1756-0500-1-76 (PMC2546417; doi:10.1186/1756-0500-1-76)
Supplement: Additional File 1 — Adolescent questionnaire v1 29 Dec 2004.pdf. Self-administered questionnaire utilized in this research. [file 1756-0500-1-76-S1.pdf]

**PHRU Adolescent Vaccine Attitudes Questionnaire**

The purpose of this survey is to help us understand how to meet the needs of people who may want to join future studies of HIV vaccines. Before joining a study to test how well HIV vaccines work, people will need to understand the basic reasons for the study, how it will work, and the risks of participation.

No one is exactly sure how future studies will be conducted, or which vaccines will be tested, but some features of future vaccine studies are clear. Some people already know a lot about the vaccines that are being developed and how they might be tested. Other people have not had a chance to learn much about these things.

The next page contains statements about future vaccine studies. Some of the statements are correct and some are not. Please read each statement carefully, then put an 'X' in the box that shows whether you AGREE, DISAGREE, or are NOT SURE.

Before you begin answering the questions, take a few seconds to read the following instructions and examples on how to enter your answers

Illustrative Figure in DataFax Format

**Please answer the questions below as shown in the example on page 1.**

| | AGREE | DISAGREE | NOT SURE |
|---|---|---|---|
| 1.1) In general, vaccines are used to protect against infection | ☐ | ☐ | ☐ |
| 1.2) Preventive vaccine studies will enroll people who are HIV-positive and HIV-negative | ☐ | ☐ | ☐ |
| 1.3) An HIV vaccine could protect against HIV infection | ☐ | ☐ | ☐ |
| 1.4) An HIV vaccine could be used to cure HIV infected people | ☐ | ☐ | ☐ |
| 1.5) I would take an HIV vaccine if it was found to be protective | ☐ | ☐ | ☐ |
| 1.6) HIV vaccine research is unsafe | ☐ | ☐ | ☐ |
| 1.7) The HIV vaccine will have no effect on a participant's HIV test results | ☐ | ☐ | ☐ |
| 1.8) Some participants will get the real vaccine, and some will get a placebo (an inactive substance). | ☐ | ☐ | ☐ |
| 1.9) The HIV vaccine can infect you with HIV | ☐ | ☐ | ☐ |
| 1.10) People in a vaccine study will know whether or not they got placebo because only the vaccine will cause side effects | ☐ | ☐ | ☐ |

**<u>Interviewers</u>: When the participants have finished marking Page 2, collect their completed forms. Then read this page to the participants and answer their questions**.

Now you will find out more about future vaccine studies. Don't worry if this would change your answers on the sheet you just completed. It is important for us to understand what people know right now about vaccine studies before receiving additional information.

In the next few years, one or more vaccines may be ready for a large research study to test whether they prevent people from getting infected with HIV. Such a large study would occur only after the vaccine has been tested in several hundred people without causing serious side effects. The vaccines may, however, cause minor side effects such as a sore arm, fever, or a headache that could last for a few days.

A large study like this is the only way to learn whether a vaccine can prevent HIV infection. Thousands of people around the world who are at high risk for HIV infection will be asked to join. The study will last for about two years. People who join the study will receive 3 to 4 injections of either a vaccine or a placebo in the first six months. Half of the participants will get the vaccine, and half will get the placebo. A placebo is a harmless substance that can't hurt you, but it can't prevent HIV infection either. Although the placebo can not work against HIV, it can cause the same immediate reactions (such as soreness or fever) as the active vaccine. People who get the vaccine will be chose at random – like flipping a coin, or lottery. No one will know who got the vaccine until after the study is over.

At the end of the study, some people will have become infected with HIV because of their high risk behaviour. Scientists will see if the number of people who got infected in the vaccine group is different from the number who got infected in the placebo group. That comparison will show them whether the vaccine worked. Scientists also will follow everyone who got infected for several more years to see if the vaccine helped people who became HIV infected to live longer without getting sick from the virus.

Participants will get HIV risk reduction counseling and test for HIV infection about every 3 – 6 months. The vaccine may cause some people to test positive on the standard HIV antibody test, even though they are not really infected and will not get AIDS from the vaccine. In this study, scientist will run special tests to see if participant who test HIV antibody positive really got infected with HIV.

Study participants may face discrimination. For example, if they tell others they are in an HIV vaccine study, they may mistakenly be thought to be HIV infected or have AIDS. Also, some participants in other vaccine studies needed to have HIV tests for insurance, hospitalization, drug treatment, etc. some of these people tested HIV-positive because of the vaccine , and had to explain why. Study staff are available to help with this problem.

**<u>Do you have any questions about this information?</u>**

Please answer the next question about your willingness to enroll in a study of a vaccine to prevent HIV infection. Put an "X" in the box for the answer that is closest to how you feel.

| | **Definitely not willing** | **Probably not willing** | **Probably willing** | **Definitely willing** |
|---|---|---|---|---|
| 2.1) How willing would you be to join a study of a vaccine to prevent HIV infection if the study began tomorrow? | ☐ | ☐ | ☐ | ☐ |

The following statements mention some reasons people may have for joining an HIV vaccine study. Put an "X" in the box that shows how important each of the following would be in your decision.

| | **Not at all important** | **Slightly important** | **Somewhat important** | **Very important** |
|---|---|---|---|---|
| 3.1) People may not want to have sex with me in I'm in a study | ☐ | ☐ | ☐ | ☐ |
| 3.2) The vaccines could cause minor side effects, that could last for a few days | ☐ | ☐ | ☐ | ☐ |
| 3.3) I may have problems with travel to foreign countries if in I'm in a study | ☐ | ☐ | ☐ | ☐ |
| 3.4) There is always a chance that a vaccine could cause problems in the future | ☐ | ☐ | ☐ | ☐ |
| 3.5) I would get free counseling and HIV tests at least once every 6 months | ☐ | ☐ | ☐ | ☐ |
| 3.6) I would get a small amount of money each time I came for a visit | ☐ | ☐ | ☐ | ☐ |
| 3.7) Some people find that, after they join studies, they think a lot about HIV/AIDS | ☐ | ☐ | ☐ | ☐ |
| 3.8) I would feel like I was helping to stop the HIV epidemic | ☐ | ☐ | ☐ | ☐ |
| 3.9) A vaccine could weaken the body's ability to fight off HIV infection | ☐ | ☐ | ☐ | ☐ |
| 3.10) People may avoid me if I'm in a | ☐ | ☐ | ☐ | ☐ |

| | | | | |
|---|---|---|---|---|
| study | | | | |
| 3.11) I may be discriminated against at school if I volunteered for an HIV vaccine trial | ☐ | ☐ | ☐ | ☐ |
| 3.12) I would be doing something to honour people I know who have HIV or AIDS or have died of AIDS | ☐ | ☐ | ☐ | ☐ |
| 3.13) The vaccines would make me test HIV-positive, even if I wasn't infected | ☐ | ☐ | ☐ | ☐ |
| 3.14) I would get current information about HIV research | ☐ | ☐ | ☐ | ☐ |
| 3.15) I may get some protection against HIV infection from the vaccine | ☐ | ☐ | ☐ | ☐ |
| 3.16) People may think I have HIV or AIDS if I'm in a study | ☐ | ☐ | ☐ | ☐ |
| 3.17) I would feel like I was giving to my community | ☐ | ☐ | ☐ | ☐ |
| 3.18) I may feel more motivated to avoid risky behaviour | ☐ | ☐ | ☐ | ☐ |
| 3.19) People may think I'm at high risk of HIV or AIDS if I'm in a study | ☐ | ☐ | ☐ | ☐ |
| 3.20) I would feel like I was helping to find a vaccine that works | ☐ | ☐ | ☐ | ☐ |
| 3.21) Vaccines tested in studies may not prevent HIV infection | ☐ | ☐ | ☐ | ☐ |
| 3.22) How important would the following people's opinions be in your decision about joining a study? | | | | |
| Parents/guardian | ☐ | ☐ | ☐ | ☐ |
| Brother/sister | ☐ | ☐ | ☐ | ☐ |
| Friends | ☐ | ☐ | ☐ | ☐ |
| Your doctor/health care provider | ☐ | ☐ | ☐ | ☐ |
| Religious leader | ☐ | ☐ | ☐ | ☐ |

Now we'd like to ask you some questions about your attitudes to HIV in general. Please put an "X" in the box that is closest to how you feel.

| | |
|---|---|
| 4.1) In your lifetime, have you ever talked to anyone about HIV or AIDS? | ☐ **Yes > go to question 4.2**<br>☐ **No > go to question 4.3** |

4.2) You answered YES. Who have you spoken to about HIV/AIDS?

| | **Yes** | **No** | **Unsure** |
|---|---|---|---|
| Family members (parents, etc.) | ☐ | ☐ | ☐ |
| Friends (not related to you) | ☐ | ☐ | ☐ |
| Doctors, nurses, other health worker | ☐ | ☐ | ☐ |
| Religious leader | ☐ | ☐ | ☐ |
| Teachers | ☐ | ☐ | ☐ |
| Other, specific | ☐ | ☐ | ☐ |

| | |
|---|---|
| 4.3) Have you ever had your blood tested for HIV? | ☐ **Yes > go to question 4.5**<br>☐ **No > go to question 4.4** |

4.4) You answered NO. What were the reasons you have never had your blood tested for HIV/AIDS?

| | **Yes** | **No** |
|---|---|---|
| Don't think I'm at risk for HIV | ☐ | ☐ |
| Afraid, nervous to get tested | ☐ | ☐ |
| Don't know where to get tested | ☐ | ☐ |
| Worried people would think I'm sick | ☐ | ☐ |
| Don't have enough money for test | ☐ | ☐ |
| Don't have time or opportunity | ☐ | ☐ |
| Testing site is too far from home | ☐ | ☐ |
| Other, specific | ☐ | ☐ |

| | |
|---|---|
| 4.5) You answered YES. The most recent time you were tested for HIV/AIDS, did you receive the results of your test? | ☐ **Yes > go to question 4.7**<br>☐ **No > go to question 4.6** |

4.6) You answered NO. What were the reasons you have did not receive your blood test results?

| | **Yes** | **No** |
|---|---|---|
| Nervous, afraid to learn results | ☐ | ☐ |
| Fear of parents reaction | ☐ | ☐ |
| Didn't have time or opportunity to get results | ☐ | ☐ |
| Other, specific | ☐ | ☐ |

| | |
|---|---|
| 4.6) Now I would like you to think about the most recent time that you were tested for HIV/AIDS. Did you tell at least one person the results of your test? | ☐ **Yes**<br>☐ **No** |
| 4.6) Should your parents know your HIV test results? | ☐ **Yes**<br>☐ **No** |
| 4.6) Children aged 9 are beginning to be sexually active | ☐ **Agree**<br>☐ **Disagree** |
| 4.7) Adults should talk to children aged between 9-16 about HIV/AIDS | ☐ **Agree**<br>☐ **Disagree** |
| 4.8) Adults do talk to children aged between 9-16 about HIV/AIDS | ☐ **Agree**<br>☐ **Disagree** |
| 4.9) HIV/AIDS is a big threat for children aged between 9-16 | ☐ **Agree**<br>☐ **Disagree** |
| 4.10) In general I have confidence in medical research | ☐ **Yes**<br>☐ **No** |
| 4.11) Adults should start talking to children about sex from 9 years | ☐ **Agree**<br>☐ **Disagree** |
| 4.12) Adults do talk to children aged between 9-16 about sex | ☐ **Agree**<br>☐ **Disagree** |
| 4.13) Should your parents give permission for you to have an HIV test? | ☐ **Yes**<br>☐ **No** |
| 4.14) Vaccines in general are good in preventing diseases | ☐ **Agree**<br>☐ **Disagree** |
| 4.15) I have someone close to me who is living with HIV/AIDS | ☐ **Yes**<br>☐ **No** |
| 4.16) This influences my decision to participate in HIV-related research | ☐ **Yes**<br>☐ **No** |

Now I’m going to ask you how you feel about people with HIV/AIDS. Different people feel differently about people who have HIV/AIDS. Please try to be as honest as you can. Please tell me how strongly you agree or disagree with the following statements.

| | Strongly agree | Somewhat agree | Not sure | Somewhat disagree | Strongly disagree |
|---|---|---|---|---|---|
| 4.17) I am afraid of people with HIV/AIDS | ☐ | ☐ | ☐ | ☐ | ☐ |
| 4.18) I feel uncomfortable around people with HIV/AIDS | ☐ | ☐ | ☐ | ☐ | ☐ |
| 4.19) I would not want to be friends with a person who has HIV/AIDS | ☐ | ☐ | ☐ | ☐ | ☐ |

Lastly, we want to ask you a few questions about yourself.

| | |
|---|---|
| 5.1) What is your age in years? | ☐☐ |
| 5.2) What is your sex (Male or Female) | ☐☐☐☐☐☐ |
| 5.3) Are you attending school? (yes or no) | ☐☐☐ |

.
